# Supplementary material for: Intravenous midazolam alters short-interval paired-pulse TMS responses differently in younger and older adults
Source: PLoS One. 2026 Nov 6;21(11):e0353088. doi: 10.1371/journal.pone.0353088 (PMC13743024; doi:10.1371/journal.pone.0353088)
Supplement: Supplementary file 1 [file pone.0353088.s001.docx]

**Table S1. Characteristics of MEP trials with interpolated peak-to-peak amplitudes.**

|  |  |  | **Younger** | | | | | **Older** | | | |
| --- | --- | --- | --- | --- | --- | --- | --- | --- | --- | --- | --- |
|  |  |  | **No Drug** | **Midaz** | **Total** |  | **No Drug** | | **Midaz** | **Total** |  |
| **SP** |  | **# Trials** (%) | 27/141 (19.2) | 8/140 (5.7) | 35/281 (12.5) |  | 2/151 (1.3) | | 10/105 (9.5) | 12/256 (4.7) |  |
|  |  | **Raw MEP, µV**  (mean (SD)) | 1,275.7 (98.0) | 1,297.8 (90.1) | 1,280.8 (95.4) |  | 1,197.9 (20.9) | | 1,433.8 (51.1) | 1,394.5 (103.0) |  |
|  |  | **Interpolated MEP, µV**  (mean (SD)) | 1,374.0 (120.2) | 1,419.1 (113.0) | 1,384.3 (118.5) |  | 1,254.1 (32.5) | | 1,536.2 (114.5) | 1,489.2 (151.3) |  |
|  |  | **%∆** (SD) | 7.7 (3.8) | 9.3 (2.2) | 8.0 (3.5) |  | 4.7 (4.5) | | 7.0 (4.8) | 6.7 (4.6) |  |
| **PP** |  | **# Trials** (%) | 2/137 (1.5) | 8/137 (5.8) | 10/274 (3.7) |  | 14/146 (9.6) | | 4/120 (3.3) | 18/266 (6.8) |  |
|  |  | **Raw MEP, µV**  (mean (SD)) | 1,359.2 (39.4) | 1,343.0 (83.0) | 1,346.3 (74.7) |  | 1,230.8 (73.9) | | 1,324.1 (137.9) | 1,251.5 (95.5) |  |
|  |  | **Interpolated MEP, µV**  (mean (SD)) | 1,439.5 (86.0) | 1,450.6 (89.5) | 1,448.3 (84.1) |  | 1,323.4 (117.3) | | 1,406.6 (152.2) | 1,341.9 (126.0) |  |
|  |  | **%∆** (SD) | 5.9 (3.3) | 8.1 (3.8) | 7.6 (3.6) |  | 7.4 (4.6) | | 6.2 (3.4) | 7.1 (4.3) |  |
| **Total** |  | **# Trials** (%) | 29/278 (10.4) | 16/277 (5.8) | 45/555 (8.1) |  | 16/297 (5.4) | | 14/225 (6.2) | 30/522 (5.8) |  |
|  |  | **Raw MEP, µV**  (mean (SD)) | 1,281.5 (97.2) | 1,320.4 (86.9) | 1,295.3 (94.5) |  | 1,226.7 (69.9) | | 1,402.4 (94.0) | 1,308.7 (120.2) |  |
|  |  | **Interpolated MEP, µV**  (mean (SD)) | 1,378.5 (118.2) | 1,434.8 (99.8) | 1,398.5 (114.1) |  | 1,314.7 (112.0) | | 1,499.2 (134.6) | 1,400.8 (152.9) |  |
|  |  | **%∆** (SD) | 7.5 (3.7) | 8.7 (3.0) | 7.9 (3.5) |  | 7.1 (4.5) | | 6.8 (4.3) | 6.9 (4.4) |  |

Abbreviations: MEP, motor-evoked potential; PP, paired-pulse; SD, standard deviation; SP, single-pulse; %∆, percent change; µV, microvolts
