# Supplementary material for: Intravenous midazolam alters short-interval paired-pulse TMS responses differently in younger and older adults
Source: PLoS One. 2026 Nov 6;21(11):e0353088. doi: 10.1371/journal.pone.0353088 (PMC13743024; doi:10.1371/journal.pone.0353088)
Supplement: Supplementary file 2 [file pone.0353088.s002.docx]

Table S2. Sensitivity analysis excluding interpolated trials

| **Analysis** | **No Drug Mean (SD)** | **Midazolam Mean (SD)** | **Mean change, Midazolam − No Drug (95% CI)** | **Test** | **p** |
| --- | --- | --- | --- | --- | --- |
| **Younger adults (n=15)** | 0.776 (0.207) | 1.149 (0.546) | +0.373 (0.015 to 0.732) | paired t = 2.23 | **.042** |
| **Older adults (n=15)** | 1.221 (0.497) | 1.382 (0.648) | +0.162 (−0.219 to 0.543) | paired t = 0.91 | .377 |
| **Age difference in drug-related change** | — | — | +0.211 (−0.288 to 0.711)* | Welch t = 0.87 | .394 |
