# Supplementary material for: Intravenous midazolam alters short-interval paired-pulse TMS responses differently in younger and older adults
Source: PLoS One. 2026 Nov 6;21(11):e0353088. doi: 10.1371/journal.pone.0353088 (PMC13743024; doi:10.1371/journal.pone.0353088)
Supplement: Supplementary file 4 [file pone.0353088.s005.docx]

Point-by-point Response to Reviewers

RE: PONE-D-26-30430, entitled “Intravenous midazolam alters short-interval paired-pulse TMS responses differently in younger and older adults”

We thank the reviewers for their comments and believe we have addressed the relevant concerns. Point-by-point responses to Reviewers 2 and 3 are outlined below.

**Reviewer 2:**
*Weaknesses and critical concerns*

*Sample size and inconsistent reporting: the sample of n=15 per group (reduced to n=11 - 12 in the midazolam condition after exclusions) is modest for detecting an age-by-drug interaction, a limitation the authors themselves acknowledge. Table 1 reports N=16 per group, whereas the Results section consistently uses n=15. This discrepancy should be explicitly reconciled in the text or table legend, as it may otherwise confuse reviewers and readers.*

We thank the reviewer for pointing out a lack of clarity in the Methods. We have clarified the Methods section and added a note to the Demographics Table (Table 1) to better reflect the effects of MEP exclusions on the data.

*The use of a suprathreshold (110% RMT) conditioning stimulus departs from the classical SICI paradigm (Kujirai et al., 1993), which employs a subthreshold conditioning pulse. Although the authors transparently disclose this choice and justify it on the grounds of investigating inter-group variability, it means the results cannot be directly compared with the bulk of the SICI-benzodiazepine literature, which tempers the strength of conclusions framed around the "elimination of paired-pulse inhibition" in the context of canonical GABA physiology.*

We added language in the Methods (lines 217–219) to specify that the employed paradigm differed from the canonical SICI. We also emphasized in the limitations that future work should use a more standardized SICI paradigm.

*The study was not registered as a clinical trial, which the authors justify by its mechanistic physiology-study design. Without pre-registration, it is difficult to fully ascertain whether the stated hypotheses (including the expectation that benzodiazepine challenge would enhance SICI) were formulated a priori or post hoc - particularly given the authors' own phrasing, "Contrary to our expectation," which, while transparent, would be more persuasive if hypotheses had been pre-registered.*

We appreciate this important point. The study was designed as a mechanistic neurophysiology experiment rather than as a prospective clinical trial and therefore was not registered as a clinical trial. We agree, however, that the absence of prospective preregistration limits the ability of readers to independently verify which hypotheses were specified before examination of the data. We have revised the manuscript to make this distinction clear and have tempered language implying formal prospective hypothesis specification. In particular, we have revised the statement “Contrary to our expectation” to avoid suggesting that the hypothesis had been preregistered, and we now acknowledge the lack of prospective preregistration as a limitation.

*Midazolam dosing was standardized by body weight, but actual plasma concentrations were not measured. Given the well-documented pharmacokinetic variability of benzodiazepines in older adults (cited by the authors via Albrecht et al., 1999; Sun et al., 2008), this omission represents a meaningful interpretive limitation - appropriately flagged in the discussion, though it could have been partially addressed with even a single-timepoint plasma sample.*

We clarified the future studies section (line 486) to refer directly to the benefit of plasma sampling.

*The authors correctly identify that the combination of lower baseline MEP amplitudes in older adults and further amplitude reduction under midazolam may have constrained the dynamic range available for the paired-pulse ratio measure. This is a critical caveat to the central finding of "no measurable drug effect" in older adults - the apparent pharmacological difference.*

We have softened the Discussion (lines 430–431) language to reflect that “We did not detect an additional midazolam-related change in the paired-pulse response in older adults.”

*Consider adding a formal age × drug-condition interaction test, rather than relying solely on separate within- and between-group t-tests, which would strengthen inference about the differential drug response by age.*

We thank the reviewer for this suggestion. We added a formal age × drug-condition interaction test to directly evaluate whether the effect of midazolam differed between younger and older adults. A 2 × 2 mixed analysis with age group as the between-subject factor and drug condition as the within-subject factor showed that the age × drug-condition interaction was not significant, F(1,28) = 0.75, p=0.394, partial η^2^=0.026. The mean change in paired-pulse ratio following midazolam was 0.373 in younger adults and 0.162 in older adults, with a between-group difference in change of 0.211 (95% CI −0.288 to 0.711). We revised the Methods, Results and Discussion to distinguish the significant within-group response observed in younger adults from evidence of a statistically significant age difference in drug response, which the interaction analysis did not support.

*Seven percent of trials (75 of 1,077) required cubic spline interpolation due to amplifier clipping (1.56 mV recording range). Although the reported interpolation error is modest (7.9±3.5% in younger, 6.9±4.4% in older subjects) and appears acceptable, a sensitivity analysis excluding these interpolated trials would strengthen confidence that the conclusions are not artifacts of the interpolation procedure.*

We thank the reviewer for this suggestion. We conducted a sensitivity analysis excluding trials requiring cubic spline interpolation. We recalculated participant-level single-pulse and paired-pulse MEP amplitudes using only QC-passing, non-interpolated trials and then recomputed the paired-pulse ratios. The principal pattern of findings was retained. In younger adults, the paired-pulse ratio increased from 0.776 ± 0.207 without drug to 1.149 ± 0.546 following midazolam (mean change = 0.373, 95% CI [0.015 0.732], t(14) = 2.23, p = 0.042). In older adults, the corresponding change from 1.221 ± 0.497 to 1.382 ± 0.648 was not significant (mean change = 0.162, 95% CI [−0.219 0.543], t(14) = 0.91, p = 0.377). These results indicate that the observed within-group effects were not dependent on cubic spline reconstruction of clipped MEPs. We have added this sensitivity analysis to the revised Methods and Results, including a table of the Non-Interpolated Group Comparisons (Table S2).

*Reliance on a standard MNI template brain rather than individualized neuronavigation is a notable limitation, particularly given known age-related anatomical differences (e.g., increased coil-to-cortex distance in older adults), which could introduce a systematic between-group measurement bias.*

We added a note about using the MNI template in the paper's limitations paragraph (line 478).

*The findings contrast with earlier pharmacological TMS studies reporting increased SICI following oral benzodiazepines (diazepam, lorazepam, alprazolam). The authors reasonably attribute this to differences in drug pharmacology, route of administration, and stimulation parameters; however, the discussion could more deeply engage with the hypothesis that midazolam's shorter duration of action and distinct receptor-binding profile, relative to the longer-acting agents used in the cited studies, may underlie the divergent results.*

We thank the reviewer for this suggestion, and we have included further discussion (lines 442–447) of the pharmacological profile, pharmacodynamics, route of administration (oral vs. IV infusion), and timing in the revised manuscript.

**Reviewer #3:**

*It is well known that the degree of brain atrophy significantly influences TMS results. To prevent brain atrophy from affecting the experimental results, conducting non-invasive MRI scans to exclude subjects with functional abnormalities is key to this study. What was the reason for deliberately not performing screening via MRI?*

We agree that individual neuroimaging would have significantly enhanced targeting of cortical regions and mitigated concerns of age-related atrophy impacting spatial variability associated with use of a template brain for neuronavigation. Funding considerations precluded our employ of personalized MRI for screening and cortical targeting, as direct costs for the study were provided by limited institutional funds.

*Regarding the evaluation of the effects of midazolam, there is no mention of how many hours after administration the TMS test was conducted. Presumably, the timing was based on the estimated 30 minutes required for blood concentrations to peak; however, variations are expected due to differences in body fat mass. The time elapsed from administration to measurement should be specified.*

We thank the reviewer for highlighting the importance of more explicitly stating the experimental timeline. We obtained TMS measures immediately after the anesthesiologist confirmed administration of midazolam dose and completed an assessment of the Observer’s Assessment of Alertness/Sedation Scale (OAA/S) (in all cases within five minutes of administration). We have added appropriate language to the Methods of the manuscript and better highlighted the timing of TMS respective of midazolam administration specifically on lines 243–244.

We would like to clarify that the 30-minute peak threshold mentioned by the reviewer typically applies to oral or intramuscular administrations where an extended absorption phase is required. In our study, midazolam was administered via an intravenous bolus followed immediately by a continuous IV infusion. Because an IV bolus bypasses the absorption phase entirely, the peak plasma concentration is achieved immediately upon injection (Heizmann et al. 1983). In a two-compartment kinetic model, this central compartment concentration drops by roughly 90% over the first two hours due to rapid peripheral distribution. To counter this drop and stabilize the plasma baseline for our TMS measurements, we integrated a continuous maintenance infusion, which achieves and maintains a steady-state plasma level directly following the loading bolus (Persson et al 1987). We recognize that variations in peripheral fat absorption can alter midazolam kinetics under normal clinical scenarios. However, fat distribution does not delay the initial, near-instantaneous concentration peak inside the central compartment. By administering the loading dose intravenously and coupling it with a continuous infusion, we established a stable and rapid steady-state window, rendering absorption-related body fat variations negligible during our target testing period.
