# Supplementary material for: Intravenous midazolam alters short-interval paired-pulse TMS responses differently in younger and older adults
Source: PLoS One. 2026 Nov 6;21(11):e0353088. doi: 10.1371/journal.pone.0353088 (PMC13743024; doi:10.1371/journal.pone.0353088)
Supplement: Peer review history

# Peer review: Decision Letter 0

**DOI:** 10.1371/journal.pone.0353088.r001

**Authors:** Tomoyoshi Komiyama

10 Aug 2026

Dear Dr. McGregor,

Thank you for submitting your manuscript to PLOS ONE. After careful consideration, we feel that it has merit but does not fully meet PLOS ONE’s publication criteria as it currently stands. Therefore, we invite you to submit a revised version of the manuscript that addresses the points raised during the review process.

Please submit your revised manuscript by Oct 09 2026 11:59PM. If you will need more time than this to complete your revisions, please reply to this message or contact the journal office at plosone@plos.org. When you're ready to submit your revision, log on to https://www.editorialmanager.com/pone/ and select the 'Submissions Needing Revision' folder to locate your manuscript file.

- A letter that responds to each point raised by the academic editor and reviewer(s). You should upload this letter as a separate file labeled 'Response to Reviewers'.
- A marked-up copy of your manuscript that highlights changes made to the original version. You should upload this as a separate file labeled 'Revised Manuscript with Track Changes'.
- An unmarked version of your revised paper without tracked changes. You should upload this as a separate file labeled 'Manuscript'.

If you would like to make changes to your financial disclosure, please include your updated statement in your cover letter. Guidelines for resubmitting your figure files are available below the reviewer comments at the end of this letter.

If applicable, we recommend that you deposit your laboratory protocols in protocols.io to enhance the reproducibility of your results. Protocols.io assigns your protocol its own identifier (DOI) so that it can be cited independently in the future. For instructions see: https://journals.plos.org/plosone/s/submission-guidelines#loc-laboratory-protocols. Additionally, PLOS ONE offers an option for publishing peer-reviewed Lab Protocol articles, which describe protocols hosted on protocols.io. Read more information on sharing protocols at https://plos.org/protocols?utm_medium=editorial-email&utm_source=authorletters&utm_campaign=protocols.

As the corresponding author, your ORCID iD is verified in the submission system and will appear in the published article. PLOS supports the use of ORCID, and we encourage all coauthors to register for an ORCID iD and use it as well. Please encourage your coauthors to verify their ORCID iD within the submission system before final acceptance, as unverified ORCID iDs will not appear in the published article. *Only*  the individual author can complete the verification step; PLOS staff *cannot*  verify ORCID iDs on behalf of authors.

We look forward to receiving your revised manuscript.

Kind regards,

Tomoyoshi Komiyama, Ph.D

Academic Editor

PLOS One

Journal Requirements:

When submitting your revision, we need you to address these additional requirements.

1.Please ensure that your manuscript meets PLOS ONE's style requirements, including those for file naming. The PLOS ONE style templates can be found at

https://journals.plos.org/plosone/s/file?id=wjVg/PLOSOne_formatting_sample_main_body.pdf and

https://journals.plos.org/plosone/s/file?id=ba62/PLOSOne_formatting_sample_title_authors_affiliations.pdf

2. We note that the grant information you provided in the ‘Funding Information’ and ‘Financial Disclosure’ sections do not match.

When you resubmit, please ensure that you provide the correct grant numbers for the awards you received for your study in the ‘Funding Information’ section.

3. Please include captions for your Supporting Information files at the end of your manuscript, and update any in-text citations to match accordingly. Please see our Supporting Information guidelines for more information: http://journals.plos.org/plosone/s/supporting-information.

4. If the reviewer comments include a recommendation to cite specific previously published works, please review and evaluate these publications to determine whether they are relevant and should be cited. There is no requirement to cite these works unless the editor has indicated otherwise.

Additional Editor Comments:

Dear authors,

Your research showed that intravenous midazolam reduced corticospinal excitability in both younger and older adults,

but while it abolished baseline paired-pulse inhibition in younger adults it produced little additional effect in older adults,

suggesting that aging alters motor cortical excitability and the cortical response to benzodiazepine.

However, I think that it is necessary to strengthen the reliability of these results by adding as much information as possible.

We thus have some questions and suggestions for the manuscript that you might consider.

I believe these comments will be helpful in the revision of your study.

Tomoyoshi Komiyama

[Note: HTML markup is below. Please do not edit.]

Reviewer's Responses to Questions

**Comments to the Author**

1. Is the manuscript technically sound, and do the data support the conclusions?

Reviewer #1: Yes

Reviewer #2: Yes

Reviewer #3: Yes

**********

2. Has the statistical analysis been performed appropriately and rigorously?

Reviewer #1: Yes

Reviewer #2: Yes

Reviewer #3: Yes

**********

3. Have the authors made all data underlying the findings in their manuscript fully available?

The PLOS Data policy

Reviewer #1: Yes

Reviewer #2: Yes

Reviewer #3: Yes

**********

4. Is the manuscript presented in an intelligible fashion and written in standard English?

Reviewer #1: Yes

Reviewer #2: Yes

Reviewer #3: Yes

**********

Reviewer #1: This is an automated report for PONE-D-26-30430. This report was solicited by the PLOS One editorial team and provided by ScreenIT. ScreenIT is an independent group of scientists developing automated tools that analyze academic papers. A set of automated tools screened your submitted manuscript and provided the report below. Each tool was created by your academic colleagues with the goal of helping authors. The tools look for factors that are important for transparency, rigor and reproducibility, and we hope that the report might help you to improve reporting in your manuscript. Within the report you will find links to more information about the items that the tools check. These links include helpful papers, websites, or videos that explain why the item is important. While our screening tools aim to improve and maintain quality standards they may, on occasion, miss nuances specific to your study type or flag something incorrectly. Each tool has limitations that are described on the ScreenIT website. The tools screen the main file for the paper; they are not able to screen supplements stored in separate files. Please note that the Academic Editor had access to these comments while making a decision on your manuscript. The Academic Editor may ask that issues flagged in this report be addressed. If you would like to provide feedback on the ScreenIT tool, please email the team at ScreenIt@bih-charite.de. If you have questions or concerns about the review process, please contact the PLOS One office at plosone@plos.org.

Reviewer #2: This manuscript presents a mechanistic study combining single-pulse and paired-pulse transcranial magnetic stimulation (TMS) of the motor cortex with a pharmacological benzodiazepine challenge (intravenous midazolam) in younger (18 - 35 years) and older (50 - 69 years) healthy volunteers. The work addresses an important and underexplored question: how aging modifies the motor cortical response to intravenous benzodiazepine challenge, as captured by neurophysiological measures. The principal strength of the paper lies in its rigorous experimental design - anesthesia-level physiological monitoring, weight-adjusted midazolam dosing, and spline interpolation to handle amplifier-clipped EMG signals - together with a notably cautious and well-calibrated interpretation of the findings. The authors explicitly acknowledge that their paired-pulse measure does not constitute a direct, receptor-specific index of GABAergic inhibition, but rather reflects the net output of interacting inhibitory and facilitatory circuits.

The use of intravenous midazolam with a precise weight-adjusted bolus-plus-infusion regimen, combined with full anesthesia-level monitoring (ECG, pulse oximetry, capnography, blood pressure) and ready availability of the reversal agent flumazenil, reflects a high standard of clinical safety. The use of the Observer's Assessment of Alertness/Sedation (OAA/S) scale to track arousal is methodologically sound, although - as the authors themselves note in the limitations - monitoring was intermittent rather than continuous.

The authors consistently apply Benjamini-Hochberg correction for multiple comparisons and report 95% confidence intervals for paired-pulse ratios, strengthening the reliability of the statistical inference. Results are presented transparently, including non-significant findings (e.g., p=0.17, p=0.052), rather than selectively.

Section 4.4 (Limitations) is unusually thorough and forthright. The authors openly identify seven substantive limitations, including a potential floor effect arising from lower baseline MEP amplitudes in older adults, absence of MRI-guided neuronavigation, lack of plasma midazolam concentration measurement, and use of a non-canonical paired-pulse protocol (a 110% RMT conditioning stimulus rather than the traditional subthreshold SICI paradigm). This degree of methodological self-scrutiny substantially strengthens the credibility of the work.

The discussion avoids overreach - the authors correctly emphasize that their findings do not refute the established link between classical SICI and GABAergic physiology, but instead point to the complexity of inhibitory-facilitatory circuit interactions under the specific stimulation parameters employed.

Weakness and critical concerns

Sample size and inconsistent reporting: the sample of n=15 per group (reduced to n=11 - 12 in the midazolam condition after exclusions) is modest for detecting an age-by-drug interaction, a limitation the authors themselves acknowledge. Table 1 reports N=16 per group, whereas the Results section consistently uses n=15. This discrepancy should be explicitly reconciled in the text or table legend, as it may otherwise confuse reviewers and readers.

The use of a suprathreshold (110% RMT) conditioning stimulus departs from the classical SICI paradigm (Kujirai et al., 1993), which employs a subthreshold conditioning pulse. Although the authors transparently disclose this choice and justify it on the grounds of investigating inter-group variability, it means the results cannot be directly compared with the bulk of the SICI-benzodiazepine literature, which tempers the strength of conclusions framed around the "elimination of paired-pulse inhibition" in the context of canonical GABA physiology.

The study was not registered as a clinical trial, which the authors justify by its mechanistic physiology-study design. Without pre-registration, it is difficult to fully ascertain whether the stated hypotheses (including the expectation that benzodiazepine challenge would enhance SICI) were formulated a priori or post hoc - particularly given the authors' own phrasing, "Contrary to our expectation," which, while transparent, would be more persuasive if hypotheses had been pre-registered.

Midazolam dosing was standardized by body weight, but actual plasma concentrations were not measured. Given the well-documented pharmacokinetic variability of benzodiazepines in older adults (cited by the authors via Albrecht et al., 1999; Sun et al., 2008), this omission represents a meaningful interpretive limitation - appropriately flagged in the discussion, though it could have been partially addressed with even a single-timepoint plasma sample.

The authors correctly identify that the combination of lower baseline MEP amplitudes in older adults and further amplitude reduction under midazolam may have constrained the dynamic range available for the paired-pulse ratio measure. This is a critical caveat to the central finding of "no measurable drug effect" in older adults - the apparent pharmacological difference.

Seven percent of trials (75 of 1,077) required cubic spline interpolation due to amplifier clipping (1.56 mV recording range). Although the reported interpolation error is modest (7.9±3.5% in younger, 6.9±4.4% in older subjects) and appears acceptable, a sensitivity analysis excluding these interpolated trials would strengthen confidence that the conclusions are not artifacts of the interpolation procedure.

Reliance on a standard MNI template brain rather than individualized neuronavigation is a notable limitation, particularly given known age-related anatomical differences (e.g., increased coil-to-cortex distance in older adults), which could introduce a systematic between-group measurement bias.

The findings contrast with earlier pharmacological TMS studies reporting increased SICI following oral benzodiazepines (diazepam, lorazepam, alprazolam). The authors reasonably attribute this to differences in drug pharmacology, route of administration, and stimulation parameters; however, the discussion could more deeply engage with the hypothesis that midazolam's shorter duration of action and distinct receptor-binding profile, relative to the longer-acting agents used in the cited studies, may underlie the divergent results.

Recommendations for the Authors

Reconcile the N=16 (Table 1) versus n=15 (Results) discrepancy explicitly in the text or table footnote.

Consider adding a formal age × drug-condition interaction test, rather than relying solely on separate within- and between-group t-tests, which would strengthen inference about the differential drug response by age.

Include a sensitivity analysis excluding interpolated trials.

Expand the discussion of pharmacodynamic differences between midazolam and the longer-acting benzodiazepines used in contrasting prior studies.

Consider explicitly stating in the abstract that the paired-pulse protocol departs from canonical SICI, to prevent readers who review only the abstract from over-interpreting the results as direct evidence of altered classical GABA-mediated inhibition.

Reviewer #3: Much remains unclear regarding the effects of benzodiazepine administration on paired-pulse stimulation in the motor cortex of older adults. This study concluded that benzodiazepine-induced changes in motor cortex reactivity vary with age, as intravenous administration of midazolam resulted in the disappearance of baseline paired-pulse suppression in young adults. In contrast, almost no measurable changes were observed in older adults.

This study deserves credit for avoiding oral administration—which is prone to age-related variations—and instead using intravenous injection, thereby ensuring that drug metabolism does not significantly influence the experimental results. In particular, while the half-life tends to be longer in women, the inclusion of female participants is crucial for ensuring the accuracy of the study. On the other hand, regarding the methodology, the following two points require clarification.

Q1:

It is well known that the degree of brain atrophy significantly influences TMS results. To prevent brain atrophy from affecting the experimental results, conducting non-invasive MRI scans to exclude subjects with functional abnormalities is key to this study. What was the reason for deliberately not performing screening via MRI?

Q2:

Regarding the evaluation of the effects of midazolam, there is no mention of how many hours after administration the TMS test was conducted. Presumably, the timing was based on the estimated 30 minutes required for blood concentrations to peak; however, variations are expected due to differences in body fat mass. The time elapsed from administration to measurement should be specified.

**********

what does this mean?). If published, this will include your full peer review and any attached files.

If you choose “no”, your identity will remain anonymous but your review may still be made public.

**Do you want your identity to be public for this peer review?**  For information about this choice, including consent withdrawal, please see our Privacy Policy

Reviewer #1: No

Reviewer #2: **Yes:**  Weronika Dębowska

Reviewer #3: No

**********

[NOTE: If reviewer comments were submitted as an attachment file, they will be attached to this email and accessible via the submission site. Please log into your account, locate the manuscript record, and check for the action link "View Attachments". If this link does not appear, there are no attachment files.]

To ensure your figures meet our technical requirements, please review our figure guidelines: https://journals.plos.org/plosone/s/figures

You may also use PLOS’s free figure tool, NAAS, to help you prepare publication quality figures: https://journals.plos.org/plosone/s/figures#loc-tools-for-figure-preparation.

NAAS will assess whether your figures meet our technical requirements by comparing each figure against our figure specifications.

# Author comment: Author response to Decision Letter 1

**DOI:** 10.1371/journal.pone.0353088.r002

28 Sep 2026

Point-by-point response to review:

Reviewer 2:

Sample size and inconsistent reporting: the sample of n=15 per group (reduced to n=11 - 12 in the midazolam condition after exclusions) is modest for detecting an age-by-drug interaction, a limitation the authors themselves acknowledge. Table 1 reports N=16 per group, whereas the Results section consistently uses n=15. This discrepancy should be explicitly reconciled in the text or table legend, as it may otherwise confuse reviewers and readers.

-We thank the reviewer for pointing out a lack of clarity in the Methods. We have clarified the Methods section and added a note to the Demographics Table (Table 1) to better reflect the effects of MEP exclusions on the data.

The use of a suprathreshold (110% RMT) conditioning stimulus departs from the classical SICI paradigm (Kujirai et al., 1993), which employs a subthreshold conditioning pulse. Although the authors transparently disclose this choice and justify it on the grounds of investigating inter-group variability, it means the results cannot be directly compared with the bulk of the SICI-benzodiazepine literature, which tempers the strength of conclusions framed around the "elimination of paired-pulse inhibition" in the context of canonical GABA physiology.

-We added language in the Methods on line 219 to specify that the employed paradigm differed from the canonical SICI. We emphasized in the limitations that future work should use a more standardized SICI paradigm.

The study was not registered as a clinical trial, which the authors justify by its mechanistic physiology-study design. Without pre-registration, it is difficult to fully ascertain whether the stated hypotheses (including the expectation that benzodiazepine challenge would enhance SICI) were formulated a priori or post hoc - particularly given the authors' own phrasing, "Contrary to our expectation," which, while transparent, would be more persuasive if hypotheses had been pre-registered.

-We appreciate this important point. The study was designed as a mechanistic neurophysiology experiment rather than as a prospective clinical trial and therefore was not registered as a clinical trial. We agree, however, that the absence of prospective preregistration limits the ability of readers to independently verify which hypotheses were specified before examination of the data. We have revised the manuscript to make this distinction clear and have tempered language implying formal prospective hypothesis specification. In particular, we have revised the statement “Contrary to our expectation” to avoid suggesting that the hypothesis had been preregistered, and we now acknowledge the lack of prospective preregistration as a limitation.

Midazolam dosing was standardized by body weight, but actual plasma concentrations were not measured. Given the well-documented pharmacokinetic variability of benzodiazepines in older adults (cited by the authors via Albrecht et al., 1999; Sun et al., 2008), this omission represents a meaningful interpretive limitation - appropriately flagged in the discussion, though it could have been partially addressed with even a single-timepoint plasma sample.

-We clarified the future studies section (L447) to refer directly to the benefit of plasma sampling.

The authors correctly identify that the combination of lower baseline MEP amplitudes in older adults and further amplitude reduction under midazolam may have constrained the dynamic range available for the paired-pulse ratio measure. This is a critical caveat to the central finding of "no measurable drug effect" in older adults - the apparent pharmacological difference.

-We have softened the Discussion (L393) language to reflect that “We did not detect an additional midazolam-related change in the paired-pulse response in older adults.”

Consider adding a formal age × drug-condition interaction test, rather than relying solely on separate within- and between-group t-tests, which would strengthen inference about the differential drug response by age.

-We thank the reviewer for this suggestion. We added a formal age × drug-condition interaction test to directly evaluate whether the effect of midazolam differed between younger and older adults. A 2 × 2 mixed analysis with age group as the between-subject factor and drug condition as the within-subject factor showed that the age × drug-condition interaction was not significant, F(1,28) = 0.75, p=.394, partial η2=.026. The mean change in paired-pulse ratio following midazolam was 0.373 in younger adults and 0.162 in older adults, with a between-group difference in change of 0.211 (95% CI −0.288 to 0.711). We revised the Results and Discussion to distinguish the significant within-group response observed in younger adults from evidence of a statistically significant age difference in drug response, which the interaction analysis did not support.

Seven percent of trials (75 of 1,077) required cubic spline interpolation due to amplifier clipping (1.56 mV recording range). Although the reported interpolation error is modest (7.9±3.5% in younger, 6.9±4.4% in older subjects) and appears acceptable, a sensitivity analysis excluding these interpolated trials would strengthen confidence that the conclusions are not artifacts of the interpolation procedure.

-We thank the reviewer for this suggestion. We conducted a sensitivity analysis excluding trials requiring cubic spline interpolation. We recalculated participant-level single-pulse and paired-pulse MEP amplitudes using only QC-passing, non-interpolated trials and then recomputed the paired-pulse ratios. The principal pattern of findings was retained. In younger adults, the paired-pulse ratio increased from 0.776 ± 0.207 without drug to 1.149 ± 0.546 following midazolam (mean change = 0.373, 95% CI [0.015, 0.732], t(14) = 2.23, p = .042). In older adults, the corresponding change from 1.221 ± 0.497 to 1.382 ± 0.648 was not significant (mean change = 0.162, 95% CI [−0.219, 0.543], t(14) = 0.91, p = .377). These results indicate that the observed within-group effects were not dependent on cubic spline reconstruction of clipped MEPs. We have added this sensitivity analysis to the revised Methods and Results, including a table of the Non-Interpolated Group Comparisons (Table S2).

Reliance on a standard MNI template brain rather than individualized neuronavigation is a notable limitation, particularly given known age-related anatomical differences (e.g., increased coil-to-cortex distance in older adults), which could introduce a systematic between-group measurement bias.

-We added a note about using the MNI template in the paper's limitations paragraph (L460).

The findings contrast with earlier pharmacological TMS studies reporting increased SICI following oral benzodiazepines (diazepam, lorazepam, alprazolam). The authors reasonably attribute this to differences in drug pharmacology, route of administration, and stimulation parameters; however, the discussion could more deeply engage with the hypothesis that midazolam's shorter duration of action and distinct receptor-binding profile, relative to the longer-acting agents used in the cited studies, may underlie the divergent results.

-We thank the reviewer for this suggestion, and we have included further discussion (L435 – 448) of the pharmacological profile, pharmacodynamics, route of administration (oral vs. IV infusion), and timing in the revised manuscript.

Reviewer #3:

It is well known that the degree of brain atrophy significantly influences TMS results. To prevent brain atrophy from affecting the experimental results, conducting non-invasive MRI scans to exclude subjects with functional abnormalities is key to this study. What was the reason for deliberately not performing screening via MRI?

-We agree that individual neuroimaging would have significantly enhanced targeting of cortical regions and mitigated concerns of age-related atrophy impacting spatial variability associated with use of a template brain for neuronavigation. Funding considerations precluded our employ of personalized MRI for screening and cortical targeting, as direct costs for the study were provided by limited institutional funds.

Regarding the evaluation of the effects of midazolam, there is no mention of how many hours after administration the TMS test was conducted. Presumably, the timing was based on the estimated 30 minutes required for blood concentrations to peak; however, variations are expected due to differences in body fat mass. The time elapsed from administration to measurement should be specified.

-We thank the reviewer for highlighting the importance of more explicitly stating the experimental timeline. We obtained TMS measures immediately after the anesthesiologist confirmed administration of midazolam dose and completed an assessment of the Observer’s Assessment of Alertness/Sedation Scale (OAA/S) (in all cases within five minutes of administration). We have added appropriate language to the Methods of the manuscript and better highlighted the timing of TMS respective of midazolam administration specifically on lines 243–244.

We would like to clarify that the 30-minute peak threshold mentioned by the reviewer typically applies to oral or intramuscular administrations where an extended absorption phase is required. In our study, midazolam was administered via an intravenous bolus followed immediately by a continuous IV infusion. Because an IV bolus bypasses the absorption phase entirely, the peak plasma concentration is achieved immediately upon injection (Heizmann et al. 1983). In a two-compartment kinetic model, this central compartment concentration drops by roughly 90% over the first two hours due to rapid peripheral distribution. To counter this drop and stabilize the plasma baseline for our TMS measurements, we integrated a continuous maintenance infusion, which achieves and maintains a steady-state plasma level directly following the loading bolus (Persson et al 1987). We recognize that variations in peripheral fat absorption can alter midazolam kinetics under normal clinical scenarios. However, fat distribution does not delay the initial, near-instantaneous concentration peak inside the central compartment. By administering the loading dose intravenously and coupling it with a continuous infusion, we established a stable and rapid steady-state window, rendering absorption-related body fat variations negligible during our target testing period.

Attachment Submitted filename: PONE-D-26-30430_Response_to_Reviewers_SAS.docx

# Peer review: Decision Letter 1

**DOI:** 10.1371/journal.pone.0353088.r003

**Authors:** Tomoyoshi Komiyama

25 Oct 2026

Intravenous midazolam alters short-interval paired-pulse TMS responses differently in younger and older adults

PONE-D-26-30430R1

Dear Dr. McGregor,

We’re pleased to inform you that your manuscript has been judged scientifically suitable for publication and will be formally accepted for publication once it meets all outstanding technical requirements.

Within one week, you’ll receive an e-mail detailing the required amendments. When these have been addressed, you’ll receive a formal acceptance letter and your manuscript will be scheduled for publication.

An invoice will be generated when your article is formally accepted. Please note, if your institution has a publishing partnership with PLOS and your article meets the relevant criteria, all or part of your publication costs will be covered. Please make sure your user information is up-to-date by logging into Editorial Manager at Editorial Manager® and clicking the ‘Update My Information' link at the top of the page. For questions related to billing, please contact billing support.

If your institution or institutions have a press office, please notify them about your upcoming paper to help maximize its impact. If they’ll be preparing press materials, please inform our press team as soon as possible -- no later than 48 hours after receiving the formal acceptance. Your manuscript will remain under strict press embargo until 2 pm Eastern Time on the date of publication. For more information, please contact onepress@plos.org.

Kind regards,

Tomoyoshi Komiyama, Ph.D

Academic Editor

PLOS One

Additional Editor Comments (optional):

Dear authors,

Thank you for submitting your revised manuscript.

It was much easier to understand than the original manuscript.

I am satisfied with the responses and the edits, so I am happy to accept your study.

You have satisfactorily addressed the comments from the two reviewers.

Therefore, I have no further suggestions.

I believe this manuscript will satiate the reader's interest.

Tomoyoshi Komiyama

Reviewers' comments:

Reviewer's Responses to Questions

**Comments to the Author**

Reviewer #2: All comments have been addressed

Reviewer #3: All comments have been addressed

**********

2. Is the manuscript technically sound, and do the data support the conclusions?

Reviewer #2: Yes

Reviewer #3: Yes

**********

3. Has the statistical analysis been performed appropriately and rigorously?

Reviewer #2: Yes

Reviewer #3: Yes

**********

4. Have the authors made all data underlying the findings in their manuscript fully available?

The PLOS Data policy

Reviewer #2: (No Response)

Reviewer #3: Yes

**********

5. Is the manuscript presented in an intelligible fashion and written in standard English?

Reviewer #2: Yes

Reviewer #3: Yes

**********

Reviewer #2: All comments have been meaningfully addressed wherever possible, resulting in substantial revisions that affect the interpretation of the findings presented. Furthermore, the manuscript is well written, and the findings are clinically relevant and of interest. I therefore recommend publication.

Reviewer #3: Regarding the presence or absence of brain atrophy, I agree with the authors’ point that this could not be investigated due to budgetary constraints. Furthermore, while the administration of the drug is a factor that affects the accuracy of the paper, the authors have accurately reflected this in the Methods, Results, and Discussion sections, thereby fully satisfying the necessary and sufficient conditions for scientific rigor.

**********

what does this mean?). If published, this will include your full peer review and any attached files.

If you choose “no”, your identity will remain anonymous but your review may still be made public.

**Do you want your identity to be public for this peer review?**  For information about this choice, including consent withdrawal, please see our Privacy Policy

Reviewer #2: **Yes:**  Weronika Dębowska

Reviewer #3: No

**********

# Editor report: Acceptance letter

**DOI:** 10.1371/journal.pone.0353088.r004

**Authors:** Tomoyoshi Komiyama

PONE-D-26-30430R1

PLOS One

Dear Dr. McGregor,

I'm pleased to inform you that your manuscript has been deemed suitable for publication in PLOS One. Congratulations! Your manuscript is now being handed over to our production team.

At this stage, our production department will prepare your paper for publication. This includes ensuring the following:

* All references, tables, and figures are properly cited

* All relevant supporting information is included in the manuscript submission,

* There are no issues that prevent the paper from being properly typeset

You will receive further instructions from the production team, including instructions on how to review your proof when it is ready. Please keep in mind that we are working through a large volume of accepted articles, so please give us a few days to review your paper and let you know the next and final steps.

Lastly, if your institution or institutions have a press office, please let them know about your upcoming paper now to help maximize its impact. If they'll be preparing press materials, please inform our press team within the next 48 hours. Your manuscript will remain under strict press embargo until 2 pm Eastern Time on the date of publication. For more information, please contact onepress@plos.org.

You will receive an invoice from PLOS for your publication fee after your manuscript has reached the completed accept phase. If you receive an email requesting payment before acceptance or for any other service, this may be a phishing scheme. Learn how to identify phishing emails and protect your accounts at https://explore.plos.org/phishing.

If we can help with anything else, please email us at customercare@plos.org.

Thank you for submitting your work to PLOS One and supporting open access.

Kind regards,

PLOS One Editorial Office Staff

on behalf of

Dr. Tomoyoshi Komiyama

Academic Editor

PLOS One
